# Comparative Impact of Pharmacological Therapies on Cluster Headache Management: A Systematic Review and Network Meta-Analysis

**DOI:** 10.3390/jcm11051411

**Published:** 2022-03-04

**Authors:** Jae-Hee Kwon, Ja-Young Han, Ji-Woong Choi, Hye-Rim Park, Heeyoung Lee

**Affiliations:** Department of Clinical Medicinal Sciences, Konyang University, Nonsan 32992, Korea; kkjjhh001127@naver.com (J.-H.K.); httre555@naver.com (J.-Y.H.); wldnd9942@naver.com (J.-W.C.); u0325u@naver.com (H.-R.P.)

**Keywords:** pharmacological therapy, cluster headaches, systematic review, network meta-analysis

## Abstract

It is important to find effective and safe pharmacological options for managing cluster headache (CH) because there is limited evidence from studies supporting the general efficacy and safety of pharmacological therapies. This systematic review and network meta-analysis (NMA) analyzed published randomized controlled trials (RCTs) to evaluate the efficacy and safety of pharmacological treatments in patients with CH. The PubMed and Embase databases were searched to identify RCTs that evaluated the efficacy and safety of pharmacological treatments for CH. Efficacy outcomes included frequency and duration of attacks, pain-free rate, and the use of rescue agents. Safety outcomes were evaluated based on the number of patients who experienced adverse events. A total of 23 studies were included in the analysis. The frequency of attacks was reduced (mean difference (MD) = −1.05, 95% confidence interval (CI) = −1.62 to −0.47; *p* = 0.0004), and the pain-free rate was increased (odds ratio (OR) = 3.89, 95% CI = 2.76–5.48; *p* < 0.00001) in the pharmacological treatment group, with a lower frequency of rescue agent use than the placebo group. Preventive, acute, and triptan or non-triptan therapies did not show significant differences in efficacy (*p* > 0.05). In the NMA, different results were shown among the interventions; for example, zolmitriptan 5 mg was more effective than zolmitriptan 10 mg in the pain-free outcome (OR = 0.40, 95% CI = 0.19–0.82; *p* < 0.05). Pharmacological treatment was shown to be more effective than placebo to manage CH with differences among types of therapies and individual interventions, and it was consistently shown to be associated with the development of adverse events. Thus, individualized therapy approaches should be applied to treat CH in real-world practice.

## 1. Introduction

Cluster headache (CH) is a primary headache disorder characterized by intense headaches occurring on one side of the head and the development of cranial autonomic symptoms, including agitation, nasal congestion, and conjunctival injection [1]. If severe CH attacks are not treated, symptoms can persist for weeks to months and may even trigger suicidal ideation [2]. Although CH is rare, the significant symptoms caused by the disease have been a public health issue and a personal burden to many individuals [3]. Jensen et al. reported that >90% of CH patients experienced a negative impact on the quality of their lives, including occupational and social disabilities, during the cluster period [4]. 

However, the current understanding of the pathophysiological mechanisms of CH remains far from complete in terms of neurovascular and chronobiological aspects despite many studies investigating pathophysiological mechanisms for developing therapies to treat CH [1,5,6,7]. Due to considerable limitations in non-drug treatment [2], the American Headache Society guidelines have recommended several pharmacological treatments [1,5,6]. Nevertheless, more evidence is needed to support the efficacy of some medications for treating CH in clinical practice [1]. Relevantly, Remahl et al. reported that the rates of placebo response, such as cessation of headache attacks, were 7% to 43% in previous trials involving CH patients [8]; thus, more empirical studies are needed to demonstrate the efficacy and safety of pharmacological treatments compared to placebo. 

Furthermore, although Francis et al. previously offered a systematic review of pharmacological options to treat CH patients, the authors could not conduct quantitative analysis given the limited number of studies included and indicated that not all medications could be recommended to treat CH patients due to insufficient evidence [9]. Moreover, several current systematic reviews and meta-analyses could not investigate a wide range of pharmacological options or provide statistically powered evidence supporting CH treatment due to the scarcity of studies enrolling CH patients [10,11]. As Brandt et al. [12] reported that for clinicians, challenges in determining the relative merit of various pharmacological alternatives for treating CH and choosing the best treatments still exist, which could be solved by network meta-analysis with the same approaches as the current study. Along with controversy and limited evidence to support pharmacological treatments on CH, for the many different types of pharmacological treatments for CH, including preventive, acute, or triptans, sufficient evidence quantitatively evaluating efficacy and safety based on pharmacological treatment types has not been accumulated for treating CH patients [13]. Because, along with advantages of systematic reviews, combining the direct and indirect could provide refined estimates [14,15], we conducted a systematic review and network meta-analysis to evaluate the efficacy and safety of pharmacological options followed by subgroup analysis determining best treatments for CH care.

## 2. Materials and Methods

This systematic review and network meta-analysis was performed in accordance with the Preferred Reporting Items for Systematic Reviews and Meta-Analyses (PRISMA) guidelines [16]. The protocol was registered on the International Prospective Register of Systematic Reviews database under no. CRD42022301178.

### 2.1. Data Sources and Search Strategy

A comprehensive strategy was used to search PubMed and Embase literature databases for relevant systematic studies addressing pharmacological treatment in patients with CH. A database search was performed to identify relevant articles published up to 8 January 2021, using CH-related keywords and medical subject headings (MeSH) terms. The reference lists from other relevant articles were manually searched to identify additional potentially eligible studies. Titles and abstracts were screened using the following search terms to identify relevant texts: “cluster headache”, “histamine cephalalgia”, “ciliary neuralgia”, “Horton syndrome”, “Sluder’s neuralgia”, “sphenopalatine neuralgia”, “migraine”, “neuralgia”, “cephalgia and headache” and “RCT”. Two investigators independently searched and evaluated the articles retrieved from the databases. Discrepancies between investigators were resolved by a third investigator.

### 2.2. Study Selection

Two independent investigators evaluated the titles and abstracts of the articles retrieved in the literature search to assess their eligibility and inclusion. Randomized controlled trials (RCTs) investigating CH treatment in patients who underwent pharmacological therapy were included. All included RCTs compared pharmacological treatment with placebo. Supplements, review articles, studies published in languages other than English, and those with only a single arm were excluded. Studies with a sample size less than five and those published only as an abstract were also excluded.

### 2.3. Data Extraction and Quality Assessment

Data extracted from the included articles were as follows: year of publication, study design, type(s) of medications, aim(s) of therapy, mean age, male/female proportion, route of administration, frequency of attacks, duration of attacks, pain-free rate, and number of individuals needing rescue agents and experiencing adverse events (AEs). The risk-of-bias assessment tool developed by Cochrane Collaboration was used to evaluate the quality of the RCTs [17]. The quality of evidence was evaluated as high, normal, low, or very low according to the Grading of Recommendations, Assessment, Development and Evaluation (GRADE) approach to assess the level of confidence in each effect estimation [18].

### 2.4. Data Synthesis and Analysis

The current study assessed the efficacy and safety of pharmacological treatment in patients with CH compared with placebo. Individuals who underwent pharmacological treatment comprised the “intervention group” and those treated with placebo comprised the “placebo group”. Efficacy outcomes included frequency of attacks, duration of attacks, number of patients using rescue agents, and post-treatment pain-free rate. The safety outcome was the number of patients who experienced AEs. In addition, subgroup analysis was used to assess efficacy and safety according to the aim of therapy (i.e., preventive and acute) and compared. Depending on the aim of the treatment types, preventive treatment [13] was used to reduce the frequency of CH attacks or to restore patients to headache-free status. Acute treatment is used to provide rapid relief [13]. Another analysis in terms of treatment types as a subgroup analysis was performed to evaluate differences in the efficacy and safety between drugs with or without triptan ingredients.

#### 2.4.1. Statistical Analysis

The data used in the direct meta-analysis were analyzed using Review Manager (RevMan, Version 5.3, The Nordic Cochrane Centre, The Cochrane Collaboration: Copenhagen, Denmark, 2014) and Collaborative Meta-Analysis version 3 (Biostat Inc., Englewood, NJ, USA). Network meta-analysis was performed by either the fixed-effect or random-effect model, using the “netmeta” and “gemtc” package of R software (version 4.1.1).

#### 2.4.2. Pairwise Meta-Analysis 

The overall effect size was expressed as odds ratio (OR), and continuous outcomes were expressed as mean difference (MD), with corresponding 95% confidence intervals (CI) for comparative studies and each intervention. The I^2^ statistic was used to evaluate heterogeneity among studies, and the percentile statistics were classified as low (<25%), medium (25–50%), or high (>50%). If the resulting analysis included >10 studies, a linear regression test of the funnel plots and Egger’s test were performed to assess publication bias.

#### 2.4.3. Network Meta-Analysis

In the Bayesian framework, we performed Markov Cain Monte Carlo with 10,000 simulations in each of the 4 chains. The first 5000 simulations were considered to be the burn-in period. In each Markov chain Monte Carlo cycle, the probabilities of each treatment ranking from first to last were estimated by effect size. According to the sum of probabilities for each treatment ranking, cumulative probabilities were defined. Each treatment’s ranking was based on the calculated SUCRA (the surface under the cumulative ranking curve) values. The value of the SUCRA ranged from 0% to 100%. A higher SUCRA value represents better treatment.

#### 2.4.4. Assessment of Consistency and Heterogeneity

The net-splitting method was used to evaluate the inconsistencies between direct and indirect evidence. Differences were considered statistically significant at *p* < 0.05. Meta-regression was used to examine the quantitative influence of study characteristics on the effect size. The overall effect size was analyzed using the mean age and proportion of males at baseline included as covariates.

## 3. Results

### 3.1. Study Selection

**Figure 1:**
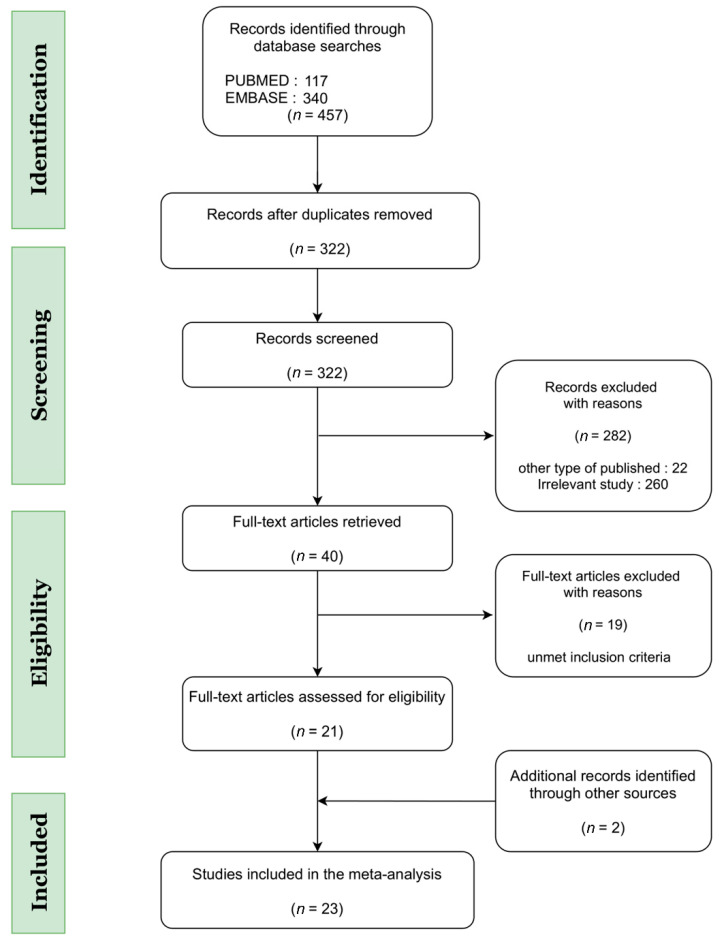
Flowchart of study identification and selection.

A comprehensive search of the PubMed and Embase databases retrieved 457 potentially relevant articles. After full-text review, however, this figure was narrowed to 40 articles, the reference lists of which were manually searched and screened to ultimately include a total of 23 studies in the present analysis (Figure 1).

### 3.2. Study Description

The basic characteristics of the 23 studies [19,20,21,22,23,24,25,26,27,28,29,30,31,32,33,34,35,36,37,38,39,40,41] included in the current investigation are summarized in Table 1. A total of 1559 patients were included in the current study. Regarding route of drug administration, twelve studies [19,23,24,25,26,27,28,30,35,37,38,41] were oral, seven [20,21,32,33,36,39,40] were injected, and four [22,29,31,34] were nasal. Zolmitriptan 5 mg (ZOL5) and zolmitriptan 10 mg (ZOL10) and placebo (PLA) were evaluated by various studies [27,34]. Galcanezumab (GAL) and PLA were evaluated by various studies [39,40]. Cimetidine (CIM) and PLA were evaluated by one study [19]. Sumatriptan 6 mg (SUM6) and PLA were evaluated by one study [20]. SUM6 and sumatriptan 12 mg (SUM12) and PLA were evaluated by one study [21]. Capsaicin (CAP) and PLA were evaluated by one study [22]. Sumatriptan 100 mg (SUM100) and PLA were evaluated by one study [23]. Melatonin (MEL) and PLA were evaluated by one study [24]. Lithium carbonate (LCAR) and PLA were evaluated by one study [25]. Misoprostol (MIS) and PLA were evaluated by one study [26]. Verapamil (VER) and PLA were evaluated by one study [28]. Civamide (CIV) and PLA were evaluated by one study [29]. Valproate (VAL) and PLA were evaluated by one study [30]. Sumatriptan nasal spray (SUMS) and PLA were evaluated by one study [31]. Octreotide (OCT) and PLA were evaluated by one study [32]. Betamethasone (BET) and PLA were evaluated by one study [33]. Frovatriptan (FRO) and PLA were evaluated by one study [35]. Cortivazol (COR) and PLA were evaluated by one study [36]. Warfarin (WAR) and PLA were evaluated by one study [37]. Candesartan cilexetil (CAN) and PLA were evaluated by one study [38]. Prednisone (PRE) and PLA were evaluated by one study [41]. Of the included studies, nine [19,20,21,26,27,31,32,34,37] were crossover designs, and fourteen [22,23,24,25,28,29,30,33,35,36,38,39,40,41] were parallel studies. Regarding the types of therapy involved, seven trials [20,21,23,27,31,34,35] investigated triptans to treat CH, and sixteen [19,22,24,25,26,28,29,30,32,33,36,37,38,39,40,41] investigated non-triptans. The therapy type consisted of thirteen preventive [19,23,24,28,29,30,33,35,37,38,39,40,41] and ten acute therapies [20,21,22,25,26,27,31,32,34,36]. The baseline characteristics of the studies, including age and male/female proportions, are summarized in Table 2.

### 3.3. Efficacy Outcomes

#### 3.3.1. Frequency of Attacks

**Figure 2:**
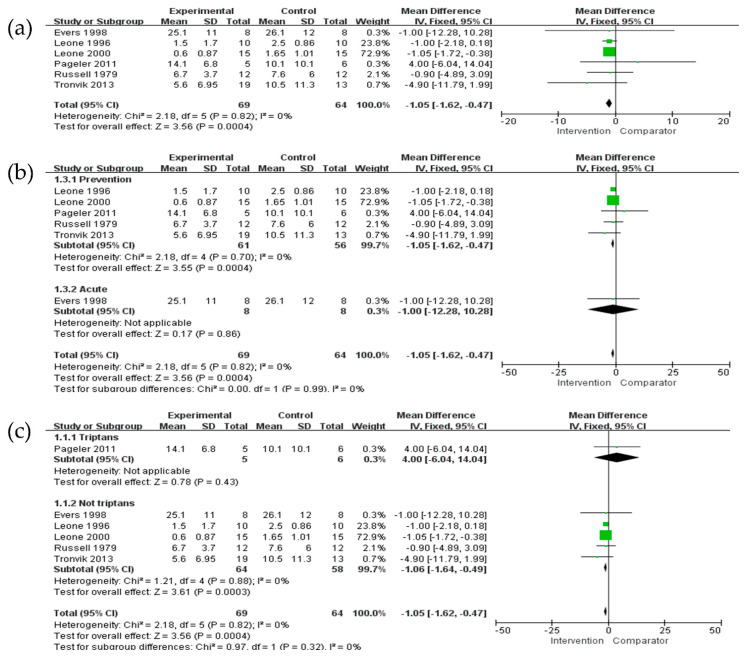
The effects of pharmacological treatments on frequency of attacks. (**a**) Overall; (**b**) comparison between preventive and acute; (**c**) comparison between triptan and non-triptan. Green squares indicated effect size for each of included studies and the size of green square indicates the weight assigned to that study in the meta-analysis. Black diamond suggested as meta-analyzed measure of effect. Bold letters represented a category or subtotal of each subgroup and overall outcome.

**Figure 3:**
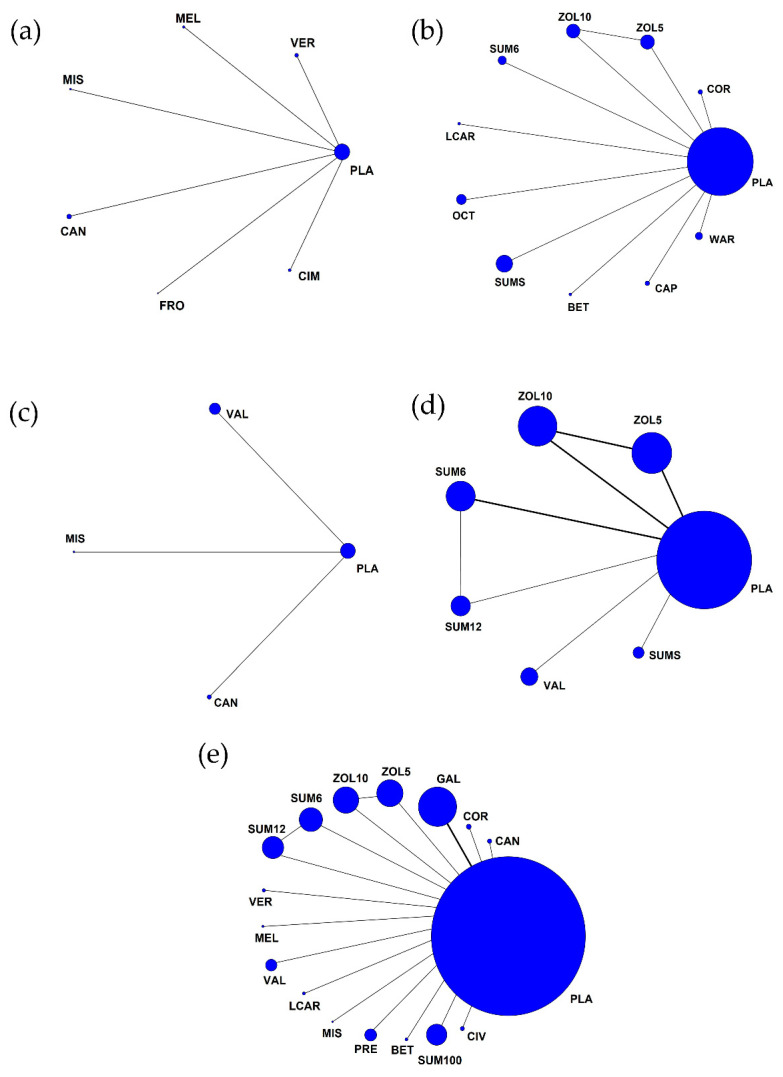
Network comparisons of studies included in the network meta-analysis. (**a**) Frequency of attacks; (**b**) pain-free rate; (**c**) duration of attacks; (**d**) number of patients using rescue agents; (**e**) adverse events. Betamethasone (BET), Candesartan cilexetil (CAN), Capsaicin (CAP), Cimetidine (CIM), Civamide (CIV), Cortivazol (COR), Frovatriptan (FRO), Galcanezumab (GAL), Lithium carbonate (LCAR), Melatonin (MEL), Misoprostol (MIS), Octreotide (OCT), Placebo (PLA), Prednisone (PRE), Sumatriptan 6 mg (SUM6), Sumatriptan 12 mg (SUM12), Sumatriptan 100 mg (SUM100), Sumatriptan spray (SUMS), Valproate (VAL), Verapamil (VER), Warfarin (WAR), Zolmitriptan 5 mg (ZOL5), Zolmitriptan 10 mg (ZOL10). The size of each circle represents the proportion of the number of patients for each treatment and the width of the lines represents the proportion of the number of studies.

**Figure 4:**
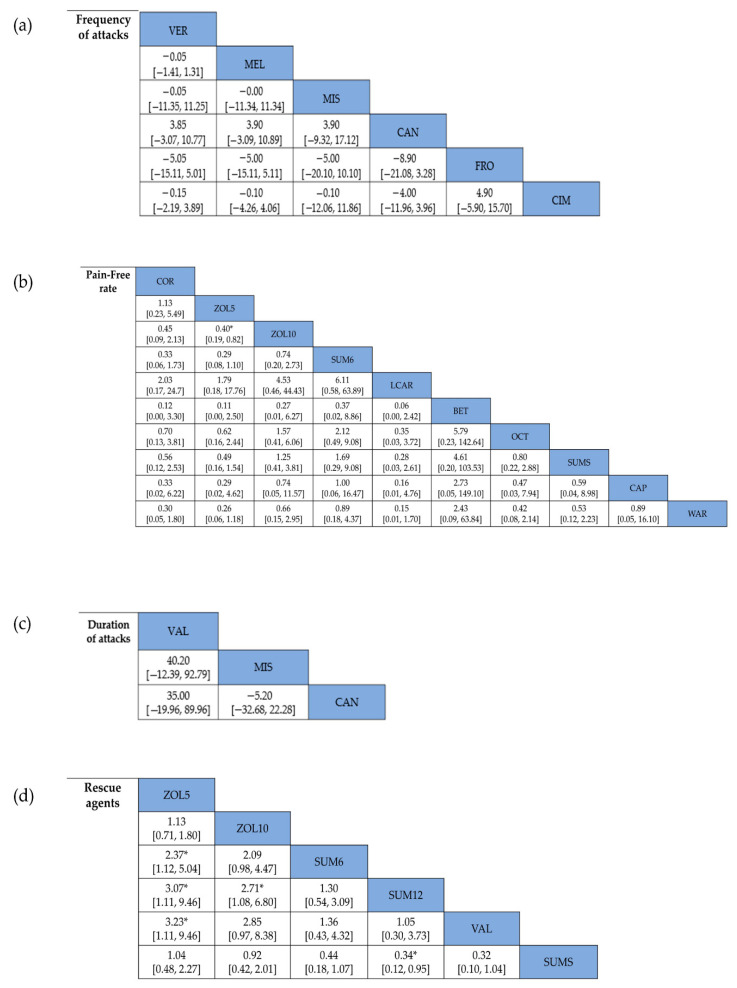

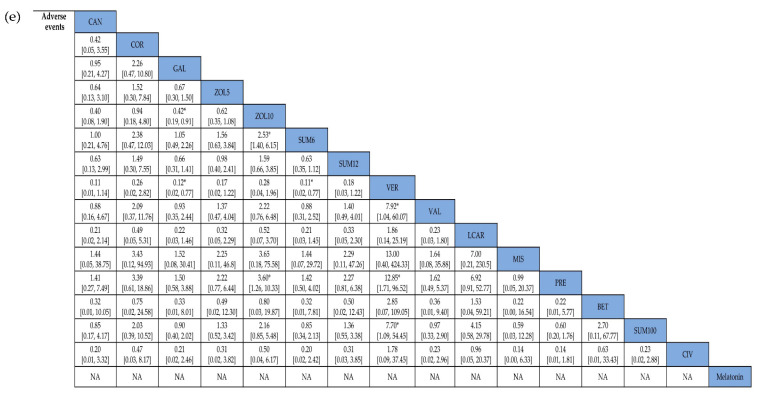
Overall network meta-analysis results of each outcome. (**a**) Frequency of attacks; (**b**) pain-free rate; (**c**) duration of attacks; (**d**) number of patients using rescue agents; (**e**) adverse events. * Statistical significance.

Six studies [19,24,26,28,35,38] reported the frequency of attacks. The overall reduction in the frequency of attacks was more significantly associated with pharmacological treatment in CH patients (MD = −1.05, 95% CI = −1.62 to −0.47; *p* = 0.0004) (Figure 2a) without significant heterogeneity. Regarding preventive treatment, five studies [19,24,28,35,38] were included in the analysis, and one study [26] was conducted with acute treatment. No difference was observed in the reduction of the frequency of attacks between preventive and acute treatments in CH patients (I^2^ = 0%, *p* = 0.99) (Figure 2b). In addition, both triptan and non-triptan drugs were associated with a reduction in the frequency of attacks in CH patients, without significant differences between the triptan and non-triptan groups (Figure 2c). The network plots of each comparison about frequency of attacks are shown in Figure 3a. In this network meta-analysis, we observed CAN use was more associated with the reduction in the frequency of attack compared to five other treatments although it was not shown in significance (*p* > 0.05). On the other hand, FRO use was less associated with decreasing the frequency of attacks to manage CH (*p* > 0.05) (Figure 4a and Supplementary Materials Figure S1).

#### 3.3.2. Pain-Free Rate

**Figure 5:**
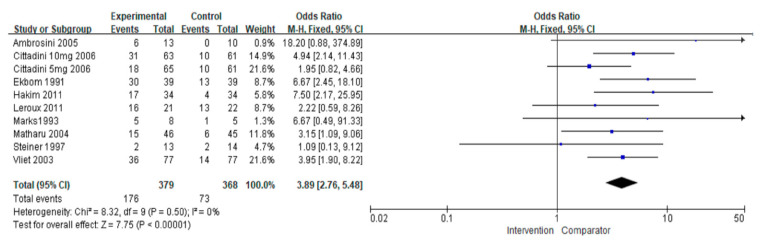
The overall effect of pharmacological treatment on pain-free rate compared to placebo. Blue squares indicated effect size for each of included studies and the size of blue square indicates the weight assigned to that study in the meta-analysis. Black diamond suggested as meta-analyzed measure of effect. Bold letters represented a category or subtotal of overall outcome.

Nine studies [20,22,25,31,32,33,34,36,37] reported pain-free rates as outcomes. The pain-free rate was higher in the intervention group (OR = 3.89, 95% CI = 2.76–5.48; *p* < 0.00001) (Figure 5). Two studies [33,37] investigated preventive treatment; seven [20,22,25,31,32,34,36] investigated acute treatment. No differences were observed between the two types of therapy, the preventive and acute treatments (I^2^ = 56.5%, *p* = 0.13). Treatment with triptans or non-triptans demonstrated a correlation with pain-free rate (OR = 3.88, 95% CI = 2.55–5.90; *p* < 0.00001 and OR = 3.90, 95% CI = 2.14–7.11; *p* < 0.00001, respectively) without differences according to the type of therapy. Heterogeneity was not observed in subgroup analyses. The network plots of each comparison about the pain-free rate are shown in Figure 3b. In the result of network meta-analysis, ZOL10 had a significantly better pain-free rate compared to ZOL5 (ZOL5 vs. ZOL10: OR = 0.40, 95% Cl = 0.19–0.82; *p* < 0.05). BET had a higher pain-free rate than nine other treatments. WAR showed a better pain-free rate than eight other treatments except the treatment BET (BET vs. WAR: OR = 2.43, 95% Cl = 0.09–63.84; *p* > 0.05). Subsequently, SUM6 had a higher pain-free rate than seven other treatments except BET (SUM6 vs. BET: OR = 0.37, 95% Cl = 0.02–8.86; *p* > 0.05) and WAR (SUM6 vs. WAR: OR = 0.89, 95% Cl = 0.18–4.37; *p* > 0.05). However, LCAR showed a lower pain-free rate than nine other treatments without showing statistical significance (*p* > 0.05) (Figure 4b and Supplementary Materials Figure S2).

#### 3.3.3. Duration of Attacks

**Figure 6:**
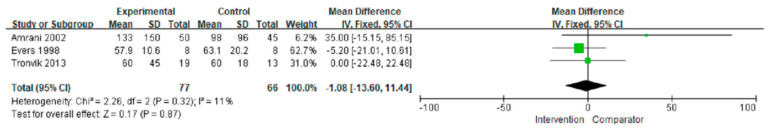
The effect of pharmacological treatment for duration of attacks compared to placebo. Green squares indicated effect size for each of included studies and the size of green square indicates the weight assigned to that study in the meta-analysis. Black diamond suggested as meta-analyzed measure of effect. Bold letters represented a category or subtotal of overall outcome.

Analysis of the duration of attacks was performed in three studies [26,30,38]. In the intervention group, a decreased duration of attacks was associated with pharmacological treatment (MD = −1.08, 95% CI = −13.60 to 11.44; *p* = 0.87) (Figure 6). The network plots of each comparison about duration of attacks are shown in Figure 3c. In our results, we observed that MIS could decrease the duration of attacks better than two other treatments (VAL vs. MIS: MD = 40.20, 95% CI = −12.39 to 92.79; MIS vs. CAN: MD = −5.20, 95% CI = −32.68 to 22.28; all *p* > 0.05). On the contrary, VAL showed fewer decreases than other treatments (Figure 4c and Supplementary Materials Figure S3). 

#### 3.3.4. Number of Patients Using Rescue Agents 

**Figure 7:**
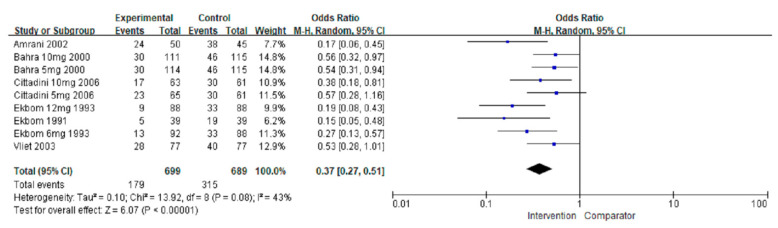
The overall effect of pharmacological treatment for the number of patients using rescue agents compared to placebo. Blue squares indicated effect size for each of included studies and the size of blue square indicates the weight assigned to that study in the meta-analysis. Black diamond suggested as meta-analyzed measure of effect. Bold letters represented a category or subtotal of overall outcome.

Analysis of the number of patients using rescue agents was performed in six studies [20,21,27,30,31,34]. An increased number of rescue agents used was associated with placebo treatment (OR = 0.37, 95% CI = 0.27–0.51; *p* < 0.00001) (Figure 7). A greater number of individuals needing rescue agents was associated with preventive treatment (OR = 0.17, 95% CI = 0.06–0.45; *p* = 0.0004) compared with acute treatment (OR = 0.41, 95% CI = 0.32–0.52; *p* < 0.00001). Comparison of triptan and non-triptan groups revealed that triptan use was associated with a lower number of rescue agent use in the intervention group than in the placebo group (OR = 0.40, 95% CI = 0.29–0.54; *p* < 0.00001) without significant differences observed in triptans (*p* = 0.10). Figure 3d shows the network plots of each comparison about number of patients using rescue agents. Compared with SUM12, SUM6 increased the number of patients using rescue agents (SUM6 vs. SUM12: OR = 1.30, 95% CI = 0.54–3.09; *p* > 0.05), but SUM6 decreased the number of patients using rescue agents when compared with ZOL10 (ZOL10 vs. SUM6: OR = 2.09, 95% CI = 0.98–4.47; *p* > 0.05). SUM12 decreased the use of rescue agents compared to ZOL10 with significant differences (ZOL10 vs. SUM12: OR = 2.71, 95% Cl = 1.08–6.80; *p* < 0.05). ZOL5 increased the number of rescue agents used compared to other treatments (Figure 4d and Supplementary Materials Figure S4).

### 3.4. Adverse Events

**Figure 8:**
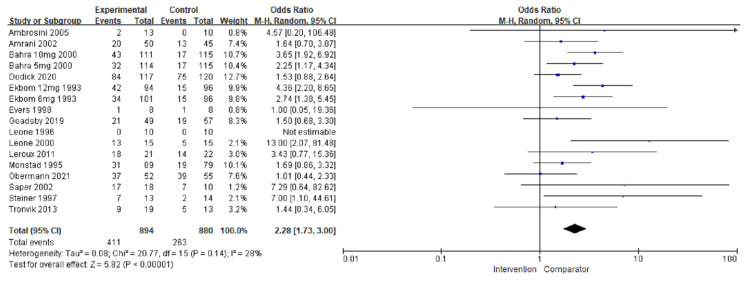
The overall effect of pharmacological treatment for the number of patients with adverse events compared to placebo. Blue squares indicated effect size for each of included studies and the size of blue square indicates the weight assigned to that study in the meta-analysis. Black diamond suggested as meta-analyzed measure of effect. Bold letters represented a category or subtotal of overall outcome.

Analysis of the number of patients who experienced AEs was performed in 15 studies [21,23,24,25,26,27,28,29,30,33,36,38,39,40,41]. In the intervention group, a greater number of patients experienced AEs compared with the placebo group (OR = 2.28, 95% CI = 1.73–3.00; *p* < 0.00001) (Figure 8), without heterogeneity. Compared with preventive treatment, a greater number of patients treated with acute pharmacological therapies experienced AEs (I^2^ = 88.6%, *p* = 0.003). In the subgroup analysis, the triptan group had a greater number of patients with AEs in the treatment group than in the control group (OR = 2.79, 95% CI = 2.07–3.76; *p* < 0.00001). Similarly, in the non-triptan group, the number of patients who experienced AEs in the treatment group was greater than that in the control group (OR = 1.78, 95% CI = 1.30–2.44; *p* = 0.0003). A greater number of patients experienced AEs in the triptan group than in the non-triptan group (*p* = 0.04). Once again, the analysis revealed no heterogeneity. The network plots of each comparison about adverse events are shown in Figure 3e. According to the network meta-analysis, SUM6 decreased the number of patients who experienced AEs compared with ZOL10 with significant differences (ZOL10 vs. SUM6: OR = 2.53, 95% Cl = 1.40–6.15; *p* < 0.05). PRE experienced a smaller number of patients with adverse events than fourteen other treatments except MIS (MIS vs. PRE: OR = 0.99, 95% CI = 0.05–20.37; *p* > 0.05). On the other hand, BET indicated a greater number of patients who experienced adverse events than other treatments (Figure 4e and Supplementary Materials Figure S5).

### 3.5. Rank Probability and SUCRA

**Figure 9:**
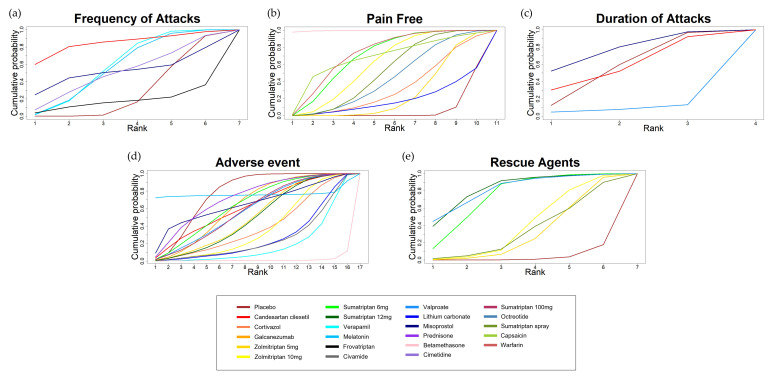
Cumulative rank with SUCRA (the surface under the cumulative ranking curve) for each treatment. The probability of each treatment distribution is displayed on the graph. (**a**) Frequency of attacks; (**b**) pain-free rate; (**c**) duration of attacks; (**d**) rescue agents; (**e**) adverse events.

The cumulative rank probabilities of each treatment are ranked in Figure 9, and SUCRA results based on the efficacy and safety outcomes are provided in Supplementary Tables S1–S5. Using the Bayesian network analysis, the SUCRA results showed the rank probabilities of all treatments from best treatment effect to the worst. Treatments with a larger area in Figure 9 were associated with larger probabilities of better outcomes. The results suggested that in the aspect of efficacy, CAN reduced the frequency of attacks the most, with a SUCRA value of 84%, while FRO reduced the frequency of attacks the least, with a SUCRA value of 18% (Supplementary Materials Table S1). BET had the highest pain-free rate, with a SUCRA value of 99%, while LCAR had the lowest, with a SUCRA value of 19%, except placebo (Supplementary Materials Table S2). MIS decreased the duration of attacks the most, with a SUCRA value of 77%, and SUM12 showed the highest value of SUCRA for the number of patients using rescue agents (83%) (Supplementary Materials Tables S3 and S4). In the aspect of safety, MEL had the smallest number of patients with adverse events and had the highest SUCRA value (76%), while BET had the greatest number of patients with adverse events and had the lowest SUCRA value (1%), except placebo (Supplementary Materials Table S5).

### 3.6. Risk of Bias and Quality of Evidence

**Figure 10:**
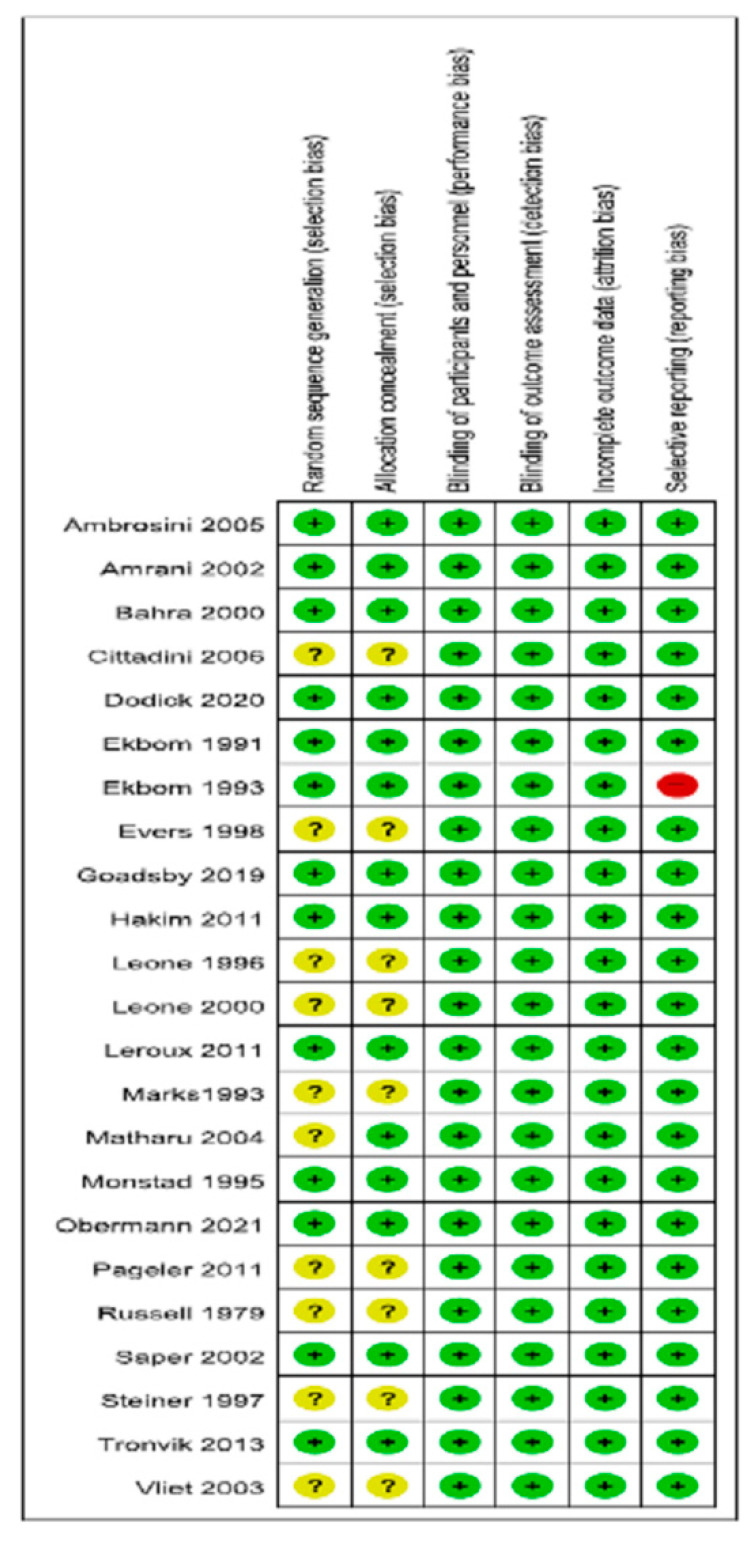
Risk-of-bias assessment of the studies. High risk of bias was marked as red (−), low risk of bias was marked as green (+) and unclear risk of bias was marked as yellow (?).

**Figure 11:**
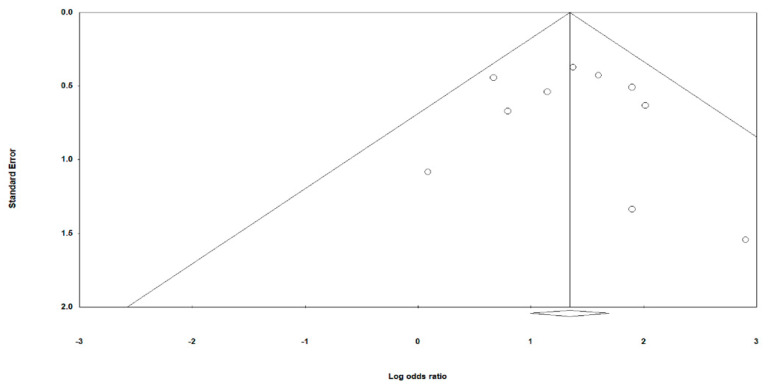
Publication bias of the studies analyzed.

The assessment of the risk of bias among the included studies is shown in Figure 10. The risk of selection bias was not clear in ten studies [19,22,24,25,26,28,31,32,34,35] and one study demonstrated a high risk of bias in terms of reporting [21]. A low risk of bias in terms of selection, performance, detection, consumption, and reporting was found in the remaining studies [20,23,27,29,30,33,36,37,38,39,40,41]. Egger’s regression test showed no evidence of publication bias (*p* = 0.75) (Figure 11). The quality of evidence according to the GRADE approach with regard to the effects of interventions for CH is summarized in Table 3.

### 3.7. Meta-Regression Analysis

Male proportion (coefficient = −6.71, 95% CI = −14.21 to 0.79; *p* = 0.08) and age (coefficient = 0.17, 95% CI = −0.02 to 0.36; *p* = 0.08) did not significantly affect the pain-free rate. Moreover, male proportion (coefficient = 1.35, 95% CI = −13.47 to 16.17; *p* = 0.89) and age (coefficient = −0.016, 95% CI = −0.19 to 0.15; *p* = 0.85) were not associated with an increase or decrease in the use of rescue agents. Male proportion (coefficient = 3.65, 95% CI = −0.41 to 7.71; *p* = 0.08) and age (coefficient = −0.08, 95% CI = −0.17 to 0.01; *p* = 0.09) did not significantly influence the effect of pharmacological treatment on CH in AEs (Supplementary Materials Figure S6).

## 4. Discussion

The current systematic review and meta-analysis aimed to compare pharmacological treatment with placebo in CH patient care. According to our results, the use of pharmacological therapies is an effective option to treat CH. Compared to placebo use, the present study demonstrated that the use of pharmacological treatment in CH patients was associated with reduced frequency and duration of attacks. Frequent severe headache attacks—likely associated with severe CH pain—impaired patient quality of life or restricted activities of daily living, which led to losses of employment and economic burdens [3]. According to Palacios Ceña et al. [42], frequent headaches negatively impact patient health, resulting in increased headache intensity and psychiatric disturbances, sometimes causing depression. Sohn et al. consistently reported that clinical factors, such as increased duration and number of pain attacks, were closely associated with disability in CH patients [43]. Considering frequency of attacks as one of important measures for prevention in CH treatment, as the present study showed, pharmacological therapy should be first engaged in the treatment of CH patients [12,44]. Moreover, previously, a systematic review and meta-analysis also demonstrated the efficacy of pharmacologic treatment, using galcanezumab, in migraine and CH in reducing headache frequency compared with placebo [11]. Although the systematic review and meta-analysis provided quantitative evidence for using pharmacological treatment instead of placebo, the study included too limited a number of CH patients to provide confidence in the evidence in practice. Furthermore, Probyn et al. also showed non-pharmacological self-management was not associated with reducing headache frequency (standard mean difference = −0.07, 95% CI = −0.22 to 0.08) [45]. Individually, the current study showed that CAN was more effective than other treatments in reducing the frequency of attacks. Etminan et al. also reported that angiotensin II receptor antagonists including candesartan reduce the frequency of headache [46]. Without comparison between medicines in the previous study and a limited number of studies analyzed, the current results should be cautiously interpreted. Because considering evidence from active-controlled trials without comparison to placebo verifying the rationality of accepted criteria is regarded as secondary evidence [12], the current study might be a supportive suggestion of pharmacological treatment for CH patient care in real-world practice.

In addition, the current study demonstrated that pharmacological therapies reduced the need for rescue agents in CH patients, providing information regarding adequate pain control [47,48]. Overuse of rescue medications for relieving headache has been frequently reported in previous studies [49,50], but increased numbers of acute medications as rescue therapy are rather associated with developing chronic headache or medication overuse headache [51]. With prior research investigating methods to reduce acute medication use in headache treatment, decreased need for rescue agents resulting from proper pharmacological treatments may represent an improvement in adherence to CH therapy as well; this displays a close association with the discontinuation of therapy [11]. Seeing as the United States Food and Drug Administration also indicated that rescue medication use should be considered an endpoint in studies investigating pain management, appropriate pharmacological therapies might contribute to reducing the number of rescue agents used by CH patients [47,48]. 

For more effective individualized medication regimens, in the current analysis, pharmacological treatment is subdivided according to the aims of therapy or active ingredients. Along with analyzing discrepancies among therapies subdivided with aims or active ingredients to treat CH patients, the current study did not demonstrate significant differences of efficacy according to the subdivisions. For recommending appropriate pharmacological therapies, prior studies reported different levels of evidence for the efficacy of therapeutics based on the aims of studies, which could not draw consistent conclusions through literature reviews [12,13]. Furthermore, although a prior meta-analysis demonstrated the efficacy of therapies including triptans as active ingredients to treat CH, the analysis only compared to placebo without comparison between therapies [52]. Although comparison between triptans and non-triptans showed no differences, among types of triptans, different efficacies were observed in types such as sumatriptans and zolmitriptans for reducing rescue agents or increasing the pain-free rate. As Pomeroy et al. also reported different efficacies among medicines including triptans, we need to consider different triptan use according to the patients’ statuses [53]. In contrast, the occurrence of AEs was differently associated with classifications depending on the treatment aims and active ingredients in the current study. Dodick et al. also reported, based on the aims of treatment, that acute treatment was more associated with the occurrence of AEs than prevention or transitional treatments with insistent needs to control symptoms due to the rapid onset [13]. Nevertheless, frequent daily dosing for treating attacks may lead to overmedication or toxicity [13], which may be associated with an increased number of individuals experiencing AEs. However, as the majority of CH patients received both preventive and acute types of medications [44], more patient-specific therapies should be applied in the practical realm. Furthermore, Law et al. showed AEs were more common with triptans than with placebo in the care of CH [52] consistent with the current study; therefore, depending on the patient’s clinical needs, a trial-and-error approach should be attempted using available pharmacological therapies in CH management [12,44].

The current study had several limitations. First, it did not evaluate the cost-effectiveness of pharmacological treatments compared with placebo in treating patients with CH. Although cost-effectiveness evaluation of therapies is an important issue, it was beyond the scope of the present study; therefore, further studies are needed. Second, the main issue of the current study is the lack of studies included to analyze differences of subgroups among aims and active ingredients. Although CH is a rare disease, complicating the ability to enroll patients to accomplish trials, less than two studies in one subgroup in the analysis could limit clinical utility. Thus, results evaluated according to subgroup classifications based on aims or active ingredients in the present study should be cautiously applied in practice; therefore, future studies in various clinical settings are expected to evaluate various clinical parameters. Third, the present study did not evaluate the sex differences related to pharmacological efficacy and safety. Although previous research has indicated different tendencies of CH development according to sex, studies included in the current analysis did not provide outcomes according to sex. Despite the scarcity of information, the current study revealed an insignificant correlation between efficacy or safety outcomes and male sex, which is consistent with reports describing a decreasing male predominance in CH development [12,54,55]. Fourth, the current study did not include head-to-head comparative trials among interventions. Considering advantages of systematic review and network meta-analysis such as generalizability and providing estimates [14,15], the current study could provide supportive evidence in clinical practice. However, to provide more precise outcomes in decision making, more future comparative studies among interventions are expected. Finally, studies that evaluated oxygen to manage CH patients were not included in the current analysis. As previous literature provided evidence related to the efficacy of oxygen treatment in CH patients [56], oxygen should be considered as an additional treatment for CH care. However, because of the controversies surrounding the inclusion of oxygen as a drug [57,58,59], we could not include studies with oxygen, and we expect more future studies evaluating oxygen as CH treatment.

## 5. Conclusions

The results of the present systematic review and network meta-analysis demonstrated that pharmacological treatments were significantly associated with a reduction in the frequency and duration of attacks and need for rescue agents and an increased pain-free rate in CH patients compared with placebo. In the subgroup analysis, there were no differences of efficacy according to treatment aims and active ingredients. However, pharmacological treatments were associated with an increase in AEs in patients with CH, especially in acute treatment and medications containing triptans. Based on the efficacy and safety of pharmacological therapies, individualized therapies should be applied to treat CH in real-world practice. 

## Figures and Tables

**Table 1:** Characteristics of the included studies.

Study Name	Publication Year	No. of Patients	Aims of Therapy	Types of Medications		Routes of Administration	Study Design
				Intervention	Comparator		
Russell et al. [19]	1979	12	prevention	cimetidine, chlorpheniramine	placebo	PO	crossover
Ekbom et al. [20]	1991	39	acute	sumatriptan	placebo	SC	crossover
Ekbom et al. [21]	1993	134	acute	sumatriptan	placebo	SC	crossover
Marks et al. [22]	1993	13	acute	capsaicin	placebo	nasal cream	parallel
Monstad et al. [23]	1995	168	prevention	sumatriptan	placebo	PO	parallel
Leone et al. [24]	1996	20	prevention	melatonin	placebo	PO	parallel
Steiner et al. [25]	1997	27	acute	lithium carbonate	placebo	PO	parallel
Evers et al. [26]	1998	8	acute	misoprostol	placebo	PO	crossover
Bahra et al. [27]	2000	124	acute	zolmitriptan	placebo	PO	crossover
Leone et al. [28]	2000	30	prevention	verapamil	placebo	PO	parallel
Saper et al. [29]	2002	28	prevention	civamide	placebo	nasal	parallel
Amrani et al. [30]	2002	95	prevention	sodium valproate	placebo	PO	parallel
Vliet et al. [31]	2003	118	acute	sumatriptan	placebo	nasal	crossover
Matharu et al. [32]	2004	57	acute	octreotide	placebo	SC	crossover
Ambrosini et al. [33]	2005	23	prevention	betamethasone	placebo	SC	parallel
Cittadini et al. [34]	2006	92	acute	zolmitriptan	placebo	nasal	crossover
Pageler et al. [35]	2011	10	prevention	frovatriptan	placebo	PO	parallel
Leroux et al. [36]	2011	43	acute	cortivazol	placebo	SC	parallel
Hakim et al. [37]	2011	34	prevention	warfarin	placebo	PO	crossover
Tronvik et al. [38]	2013	32	prevention	candesartan cilexetil	placebo	PO	parallel
Goadsby et al. [39]	2019	106	prevention	galcanezumab	placebo	SC	parallel
Dodick et al. [40]	2020	237	prevention	galcanezumab	placebo	SC	parallel
Obermann et al. [41]	2021	109	prevention	prednisone	placebo	PO	parallel

PO, Per Oral; Nasal, nasal spray; SC, Subcutaneous injection.

**Table 2:** Baseline characteristics of age and male proportion.

Study Name	Publication Year	Mean Age (Year)		Male Proportion (%)
		Intervention Group	Control Group	
Russell et al. [19]	1979	49	-	-
Ekbom et al. [20]	1991	42 ± 10	-	79.5
Ekbom et al. [21]	1993	41 ± 9	-	86.6
Marks et al. [22]	1993	-	-	23.1
Monstad et al. [23]	1995	40 ± 10	40 ± 10	88.7
Leone et al. [24]	1996	38	34	75
Steiner et al. [25]	1997	34.5 ± 19	35.7 ± 20.8	100
Evers et al. [26]	1998	44.5	-	100
Bahra et al. [27]	2000	43.8 ± 10.9	-	86.3
Leone et al. [28]	2000	44 ± 8	43 ± 10	90
Saper et al. [29]	2002	45.1 ± 10.5	43.9 ± 16.3	89.3
Amrani et al. [30]	2002	47 ± 11.3	43.6 ± 11.5	88.4
Vliet et al. [31]	2003	43 ± 11	43 ± 11	82.2
Matharu et al. [32]	2004	40 ± 10	-	78.9
Ambrosini et al. [33]	2005	42	37.7	86.9
Cittadini et al. [34]	2006	40 ± 10	-	86.9
Pageler et al. [35]	2011	-	-	-
Leroux et al. [36]	2011	CCH: 41.3 ± 13.3 ECH: 40.0 ± 7.8	CCH: 42.8 ± 11.9 ECH: 41.9 ± 10.4	88.4
Hakim et al. [37]	2011	44.1 ± 5.1	45.2 ± 4.5	76.5
Tronvik et al. [38]	2013	42 ± 10.1	41 ± 12.1	84.4
Goadsby et al. [39]	2019	47 ± 11	45 ± 11	83
Dodick et al. [40]	2020	45.6 ± 11	44.4 ± 10.8	72.6
Obermann et al. [41]	2021	42.4 ± 11.4	40.3 ± 10.5	83.5

Age is presented as mean ± SD, CCH = chronic cluster headache, ECH = episodic cluster headache.

**Table 3:** Summary of findings for efficacy and safety outcomes comparing interventions to comparators based on the GRADE approach.

Outcomes	No. of Participants (Studies)	Limitation	Inconsistency	Indirection	Imprecision	Publication Bias	Pooled Estimates	Quality of Evidence
Efficacy outcomes								
Frequency of attacks								
Overall	133 (6)	No serious	No serious	No serious	No serious	No serious	−1.05 (−1.62, −0.47)	⊕⊕⊕⊕ High
Acute	117 (5)	No serious	No serious	No serious	No serious	No serious	−1.05 (−1.62, −0.47)	⊕⊕⊕⊕ High
Transitional	133 (1)	No serious	No serious	No serious	No serious	No serious	−1.00 (−12.28, 10.28)	⊕⊕⊕⊕ High
Triptans	11 (1)	No serious	No serious	No serious	No serious	No serious	4.00 (−6.04, 14.04)	⊕⊕⊕⊕ High
Non-triptans	122 (5)	No serious	No serious	No serious	No serious	No serious	−1.06 (−1.64, −0.49)	⊕⊕⊕⊕ High
Duration of attacks								
Overall	143 (3)	No serious	No serious	No serious	No serious	No serious	−1.08 (−13.60, 11.44)	⊕⊕⊕⊕ High
Pain-free rate								
Overall	747 (9)	No serious	No serious	No serious	No serious	No serious	3.89 (2.76, 5.48)	⊕⊕⊕⊕ High
Preventive	91 (2)	No serious	No serious	No serious	No serious	No serious	8.90 (2.85, 27.79)	⊕⊕⊕⊕ High
Acute	656 (7)	No serious	No serious	No serious	No serious	No serious	3.53 (2.46, 5.07)	⊕⊕⊕⊕ High
Triptans	482 (3)	No serious	No serious	No serious	No serious	No serious	3.88 (2.55, 5.90)	⊕⊕⊕⊕ High
Non-triptans	747 (6)	No serious	No serious	No serious	No serious	No serious	3.90 (2.14, 7.11)	⊕⊕⊕⊕ High
Number of people needing rescue agents								
Overall	1388 (6)	No serious	No serious	No serious	No serious	No serious	0.37 (0.27, 0.51)	⊕⊕⊕⊕ High
Preventive	95 (1)	No serious	No serious	No serious	No serious	No serious	0.17 (0.06, 0.45)	⊕⊕⊕⊕ High
Acute	1293 (5)	No serious	No serious	No serious	No serious	No serious	0.41 (0.32, 0.52)	⊕⊕⊕⊕ High
Triptans	1293 (5)	No serious	No serious	No serious	No serious	No serious	0.40 (0.29, 0.54)	⊕⊕⊕⊕ High
Non-triptans	95 (1)	No serious	No serious	No serious	No serious	No serious	0.17 (0.06, 0.45)	⊕⊕⊕⊕ High
Safety outcomes								
Adverse events								
Overall	1822 (16)	No serious	No serious	No serious	No serious	No serious	2.16 (1.59, 2.94)	⊕⊕⊕⊕ High
Preventive	846 (10)	No serious	No serious	No serious	No serious	No serious	1.66 (1.23, 2.23)	⊕⊕⊕⊕ High
Acute	928 (5)	No serious	No serious	No serious	No serious	No serious	3.19 (2.33, 4.39)	⊕⊕⊕⊕ High
Triptans	1010 (3)	No serious	No serious	No serious	No serious	No serious	2.79 (2.07, 3.76)	⊕⊕⊕⊕ High
Non-triptans	812 (13)	No serious	No serious	No serious	No serious	No serious	1.64 (1.21, 2.23)	⊕⊕⊕⊕ High

## Supplementary Materials

The following supporting information can be downloaded at https://www.mdpi.com/article/10.3390/jcm11051411/s1, Figure S1: Direct and indirect comparison in terms of frequency of attacks; Figure S2: Direct and indirect comparison in terms of pain-free rate; Figure S3: Direct and indirect comparison in terms of duration of attacks; Figure S4: Direct and indirect comparison in terms of duration of attacks; Figure S5: Direct and indirect comparison in terms of adverse events; Figure S6: Meta-regression results with male proportion and age. Table S1: The result of surface under the cumulative ranking curve (SUCRA) for frequency of attacks; Table S2: The result of surface under the cumulative ranking curve (SUCRA) for pain-free rate; Table S3: The result of surface under the cumulative ranking curve (SUCRA) for duration of attacks; Table S4: The result of surface under the cumulative ranking curve (SUCRA) for number of patients using rescue agents; Table S5: The result of surface under the cumulative ranking curve (SUCRA) for frequency of attacks.

## References

[1] Robbins M.S., Starling A.J. (2016). Treatment of Cluster Headache: The American Headache Society Evidence-Based Guidelines. Headache.

[2] Hoffmann J., May A. (2018). Diagnosis, pathophysiology, and management of cluster headache. Lancet Neurol..

[3] Rozen T.D., Fishman R.S. (2012). Cluster headache in the United States of America: Demographics, clinical characteristics, triggers, suicidality, and personal burden. Headache.

[4] Jensen R.M., Lyngberg A., Jensen R.H. (2007). Burden of cluster headache. Cephalalgia.

[5] Goadsby P.J., Schoenen J. (2006). Towards a definition of intractable headache for use in clinical practice and trials. Cephalalgia.

[6] Pedersen J.L., Barloese M., Jensen R.H. (2013). Neurostimulation in cluster headache: A review of current progress. Cephalalgia.

[7] Ljubisavljevic S., Zidverc-Trajkovic J. (2019). Cluster headache: Pathophysiology, diagnosis and treatment. J. Neurol..

[8] Nilsson Remahl A.I., Laudon Meyer E. (2003). Placebo response in cluster headache trials: A review. Cephalalgia.

[9] Francis G.J., Becker W.J., Pringsheim T.M. (2010). Acute and preventive pharmacologic treatment of cluster headache. Neurology.

[10] Argyriou A.A., Vikelis M. (2021). Recently available and emerging therapeutic strategies for the acute and prophylactic management of cluster headache: A systematic review and expert opinion. Expert Rev. Neurother..

[11] Yang Y., Wang Z. (2020). Different doses of galcanezumab versus placebo in patients with migraine and cluster headache: A meta-analysis of randomized controlled trials. J. Headache Pain.

[12] Brandt R.B., Doesborg P.G.G. (2020). Pharmacotherapy for Cluster Headache. CNS Drugs.

[13] Dodick D.W., Rozen T.D. (2000). Cluster headache. Cephalalgia.

[14] Papakonstantinou T., Nikolakopoulou A. (2020). In network meta-analysis, most of the information comes from indirect evidence: Empirical study. J. Clin. Epidemiol..

[15] Jansen P.J., Naci H. (2013). Is network meta-analysis as valid as standard pairwise meta-analysis? It all depends on the distribution of effect modifiers. BMC Med..

[16] Liberati A., Altman D.G. (2009). The PRISMA statement for reporting systematic reviews and meta-analyses of studies that evaluate healthcare interventions: Explanation and elaboration. BMJ.

[17] Higgins J.P., Altman D.G. (2011). The Cochrane Collaboration’s tool for assessing risk of bias in randomised trials. BMJ.

[18] Balshem H., Helfand M. (2011). GRADE guidelines: 3. Rating the quality of evidence. J. Clin. Epidemiol..

[19] Russell D. (1979). Cluster headache: Trial of a combined histamine H1 and H2 antagonist treatment. J. Neurol. Neurosurg. Psychiatry.

[20] Sumatriptan Cluster Headache Study Group (1991). Treatment of acute cluster headache with sumatriptan. N. Engl. J. Med..

[21] Ekbom K., Monstad I. (1993). Subcutaneous sumatriptan in the acute treatment of cluster headache: A dose comparison study. Acta Neurol. Scand..

[22] Marks D.R., Rapoport A. (1993). A double-blind placebo-controlled trial of intranasal capsaicin for cluster headache. Cephalalgia.

[23] Monstad I., Krabbe A. (1995). Preemptive oral treatment with sumatriptan during a cluster period. Headache.

[24] Leone M., D’Amico D. (1996). Melatonin versus placebo in the prophylaxis of cluster headache: A double-blind pilot study with parallel groups. Cephalalgia.

[25] Steiner T.J., Hering R. (1997). Double-blind placebo-controlled trial of lithium in episodic cluster headache. Cephalalgia.

[26] Evers S., Masur H. (1998). Prostaglandin analog mechanisms are not effective in refractory chronic cluster headache. Headache.

[27] Bahra A., Gawel M.J. (2000). Oral zolmitriptan is effective in the acute treatment of cluster headache. Neurology.

[28] Leone M., D’Amico D. (2000). Verapamil in the prophylaxis of episodic cluster headache: A double-blind study versus placebo. Neurology.

[29] Saper J.R., Klapper J. (2002). Intranasal civamide for the treatment of episodic cluster headaches. Arch. Neurol..

[30] El Amrani M., Massiou H., Bousser M.G. (2002). A negative trial of sodium valproate in cluster headache: Methodological issues. Cephalalgia.

[31] Van Vliet J.A., Bahra A. (2003). Intranasal sumatriptan in cluster headache: Randomized placebo-controlled double-blind study. Neurology.

[32] Matharu M.S., Levy M.J. (2004). Subcutaneous octreotide in cluster headache: Randomized placebo-controlled double-blind crossover study. Ann. Neurol..

[33] Ambrosini A., Vandenheede M. (2005). Suboccipital injection with a mixture of rapid- and long-acting steroids in cluster headache: A double-blind placebo-controlled study. Pain.

[34] Cittadini E., May A. (2006). Effectiveness of intranasal zolmitriptan in acute cluster headache: A randomized, placebo-controlled, double-blind crossover study. Arch. Neurol..

[35] Pageler L., Katsarava Z. (2011). Frovatriptan for prophylactic treatment of cluster headache: Lessons for future trial design. Headache.

[36] Leroux E., Valade D. (2011). Suboccipital steroid injections for transitional treatment of patients with more than two cluster headache attacks per day: A randomised, double-blind, placebo-controlled trial. Lancet Neurol..

[37] Hakim S.M. (2011). Warfarin for refractory chronic cluster headache: A randomized pilot study. Headache.

[38] Tronvik E., Wenecke T. (2013). Randomised trial on episodic cluster headache with an angiotensin II receptor blocker. Cephalalgia.

[39] Goadsby P.J., Dodick D.W. (2019). Trial of Galcanezumab in Prevention of Episodic Cluster Headache. N. Engl. J. Med..

[40] Dodick D.W., Goadsby P.J. (2020). Phase 3 randomized, placebo-controlled study of galcanezumab in patients with chronic cluster headache: Results from 3-month double-blind treatment. Cephalalgia.

[41] Obermann M., Nagel S. (2021). Safety and efficacy of prednisone versus placebo in short-term prevention of episodic cluster headache: A multicentre, double-blind, randomised controlled trial. Lancet Neurol..

[42] Palacios-Ceña M., Fernández-Muñoz J.J. (2017). The association of headache frequency with pain interference and the burden of disease is mediated by depression and sleep quality, but not anxiety, in chronic tension type headache. J. Headache Pain.

[43] Sohn J.H., Park J.W. (2020). Clinical factors influencing the impact of cluster headache from a prospective multicenter study. Sci. Rep..

[44] May A., Schwedt T.J. (2018). Cluster headache. Nat. Rev. Dis. Primers.

[45] Probyn K., Bowers H. (2017). Non-pharmacological self-management for people living with migraine or tension-type headache: A systematic review including analysis of intervention components. BMJ Open.

[46] Etminan M., Levine M.A. (2002). Efficacy of Angiotensin II Receptor Antagonists in Preventing Headache: A Systematic Overview and Meta-analysis. Am. J. Med..

[47] U.S. Department of Health and Human Services Guidance for Industry: Analgesic Indications: Developing Drug and Biological Products. https://www.fdanews.com/ext/resources/files/02/02-05-14-Analgesic.pdf.

[48] Kivitz A.J., Conaghan P.G. (2019). Rescue Analgesic Medication Use by Patients Treated with Triamcinolone Acetonide Extended-Release for Knee Osteoarthritis Pain: Pooled Analysis of Three Phase 2/3 Randomized Clinical Trials. Pain Ther..

[49] Chiang C.C., Schwedt T.J. (2016). Treatment of medication-overuse headache: A systematic review. Cephalalgia.

[50] Dodick D., Freitag F. (2006). Evidence-based understanding of medication-overuse headache: Clinical implications. Headache.

[51] Diener H.C., Holle D. (2018). Chronic Headache Due to Overuse of Analgesics and Anti-Migraine Agents. Dtsch. Arztebl. Int..

[52] Law S., Derry S., Moore R.A. (2013). Triptans for acute cluster headache. Cochrane Database Syst. Rev.

[53] Pomeroy J.L., Marmura M.J. (2013). Pharmacotherapy Options for the Management of Cluster Headache. Clin. Med. Insights Ther..

[54] Lund N., Barloese M. (2017). Chronobiology differs between men and women with cluster headache, clinical phenotype does not. Neurology.

[55] Bahra A.A., May A., Goadsby P.J. (2002). Cluster headache: A prospective clinical study with diagnostic implications. Neurology.

[56] Pearson S.M., Burish M.J. (2019). Effectiveness of Oxygen and Other Acute Treatments for Cluster Headache: Results From the Cluster Headache Questionnaire, an International Survey. Headache.

[57] Drescher J., Khouri A. (2021). Effectiveness of medication in cluster headache. BMC Neurol.

[58] Porta M., Granella F. (1991). Treatment of cluster headache attacks with hyperbaric oxygen. Cephalalgia.

[59] (2001). Non-Pharmacological therapy of migraine. J. Headache Pain.

