# Supplementary material for: Comparative Impact of Pharmacological Therapies on Cluster Headache Management: A Systematic Review and Network Meta-Analysis
Source: J Clin Med. 2022 Mar 4;11(5):1411. doi: 10.3390/jcm11051411 (PMC8911224; doi:10.3390/jcm11051411)
Supplement: Supplementary file 1 [file jcm-11-01411-s001.zip › jcm-1603895-supplementary.pdf]

Supplementary Material

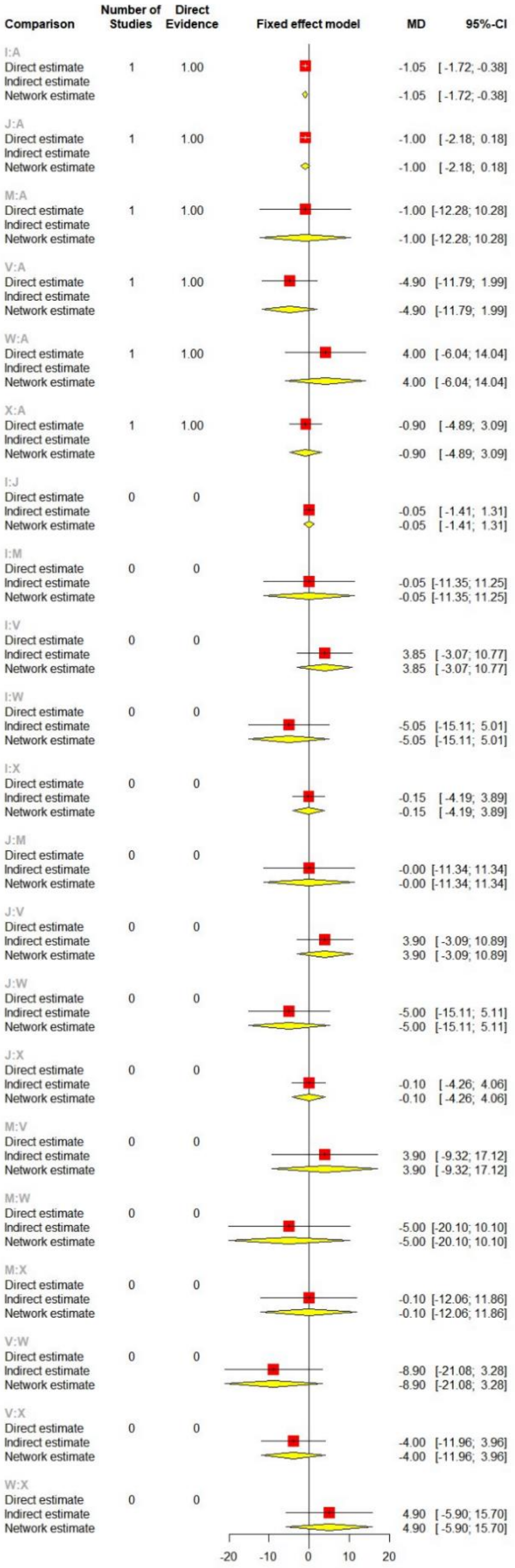

Figure S1. Direct and indirect comparison in terms of frequency of attacks.

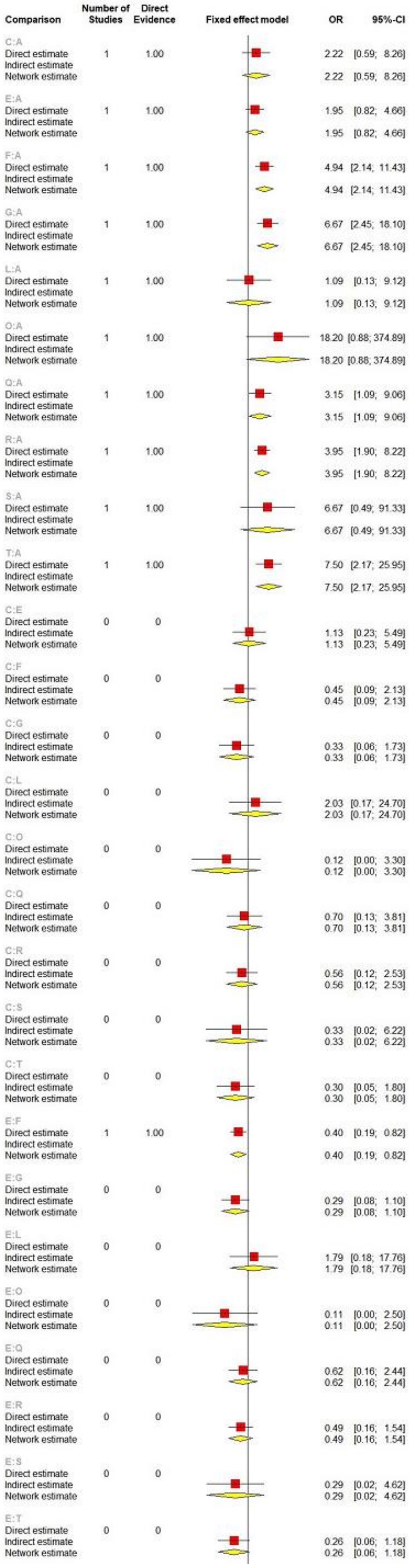

Figure S2. Direct and indirect comparison in terms of pain-free rate.

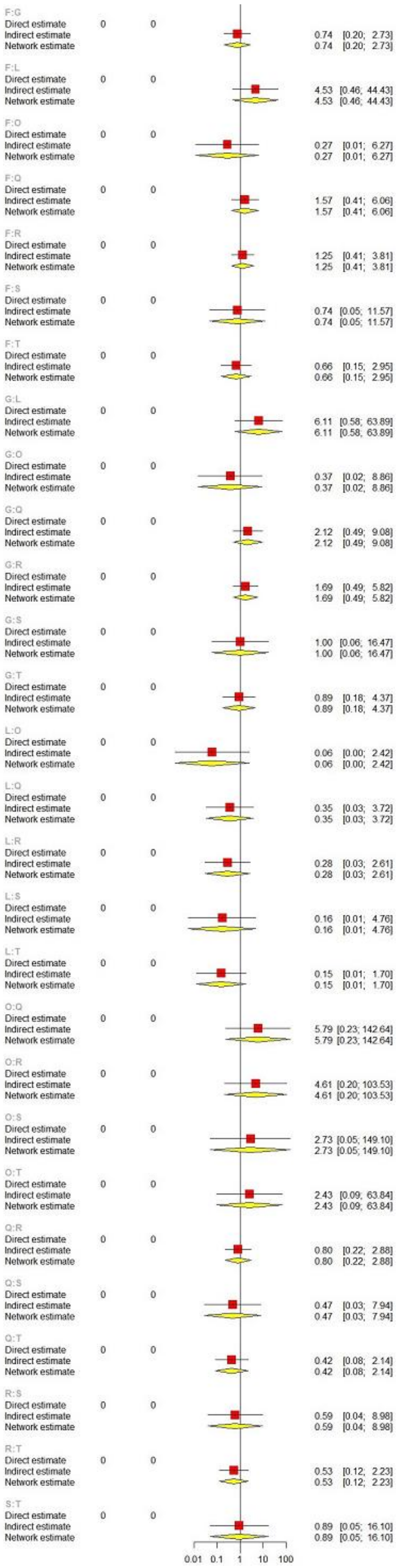

Figure S2. *cont.*

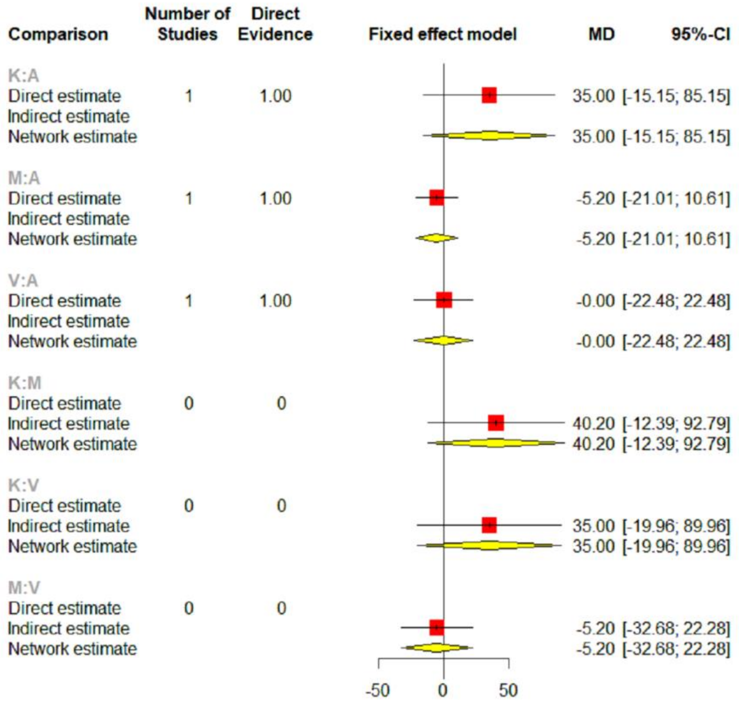

Figure S3. Direct and indirect comparison in terms of duration of attacks.

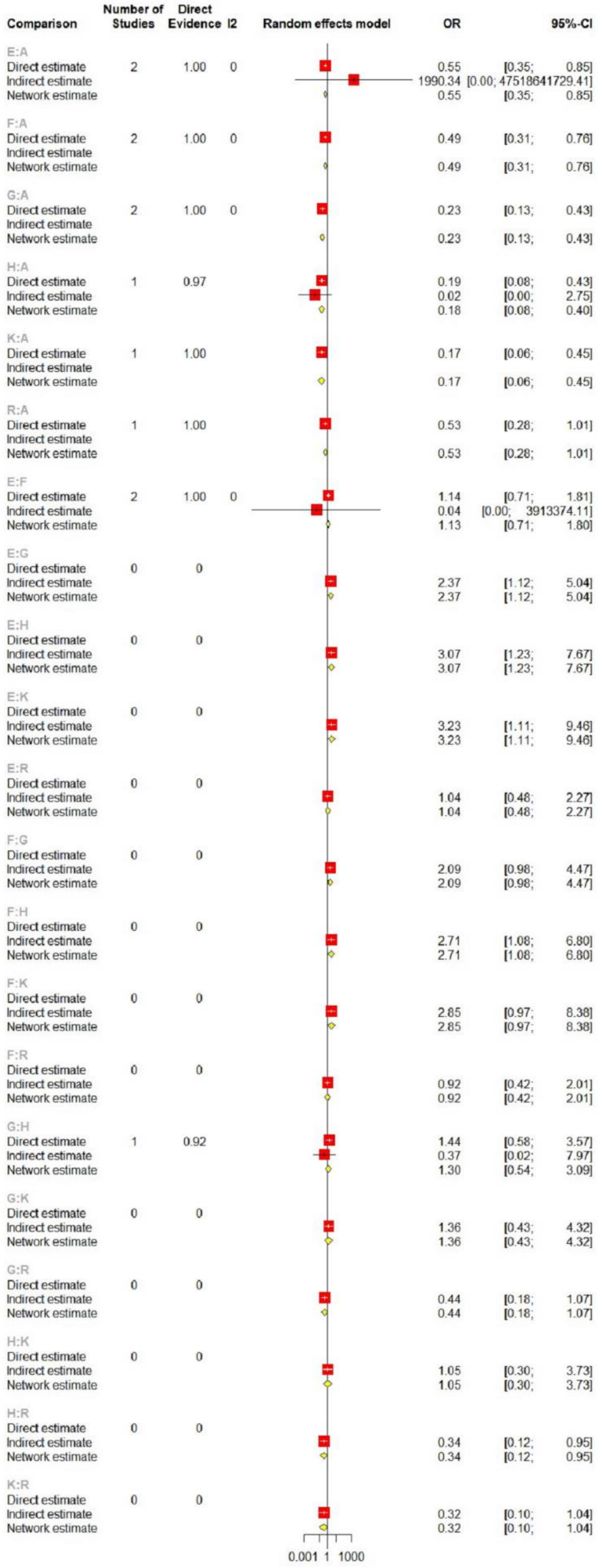

Figure S4. Direct and indirect comparison in terms of duration of attacks.

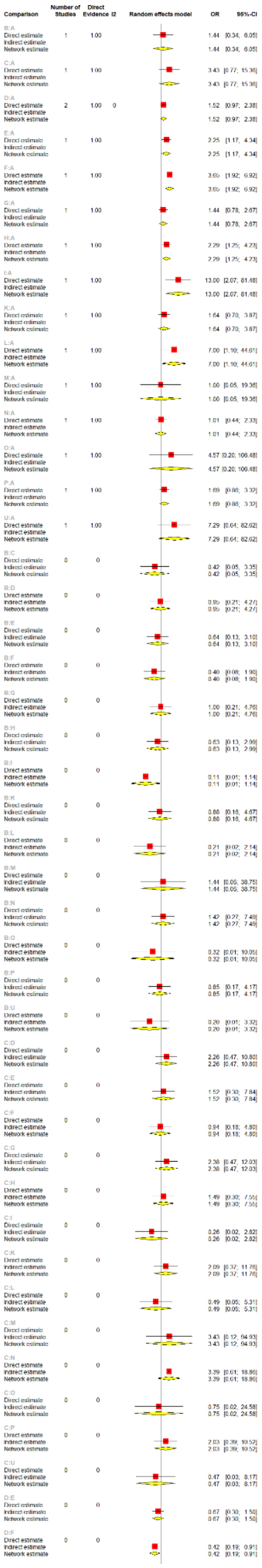

Figure S5. Direct and indirect comparison in terms of adverse events.

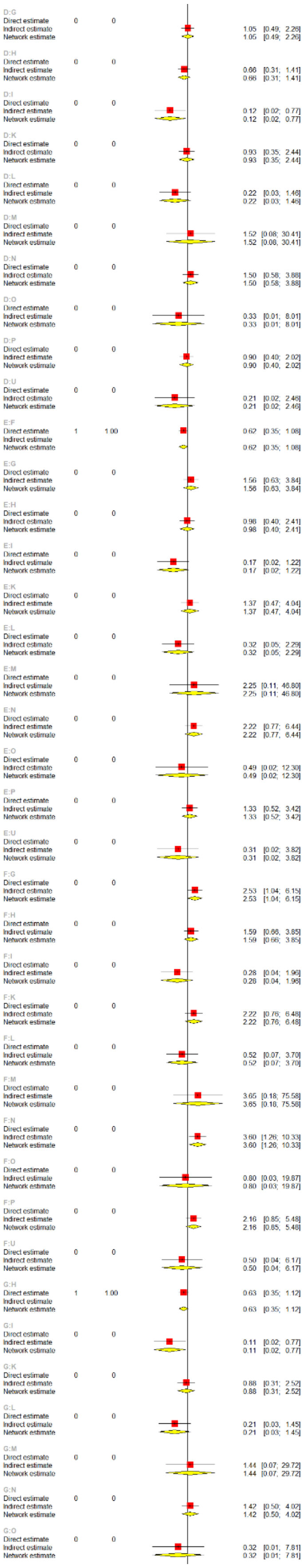

Figure S5. *cont.*

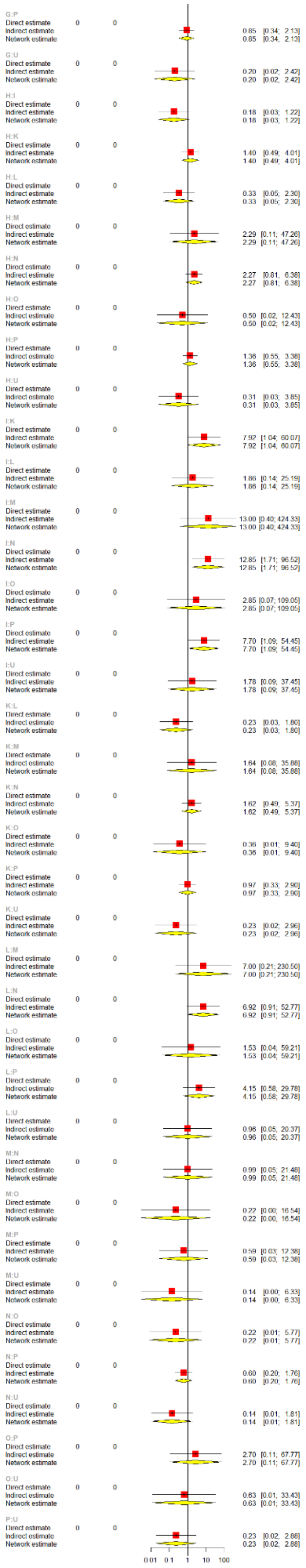

Figure S5. *cont.*

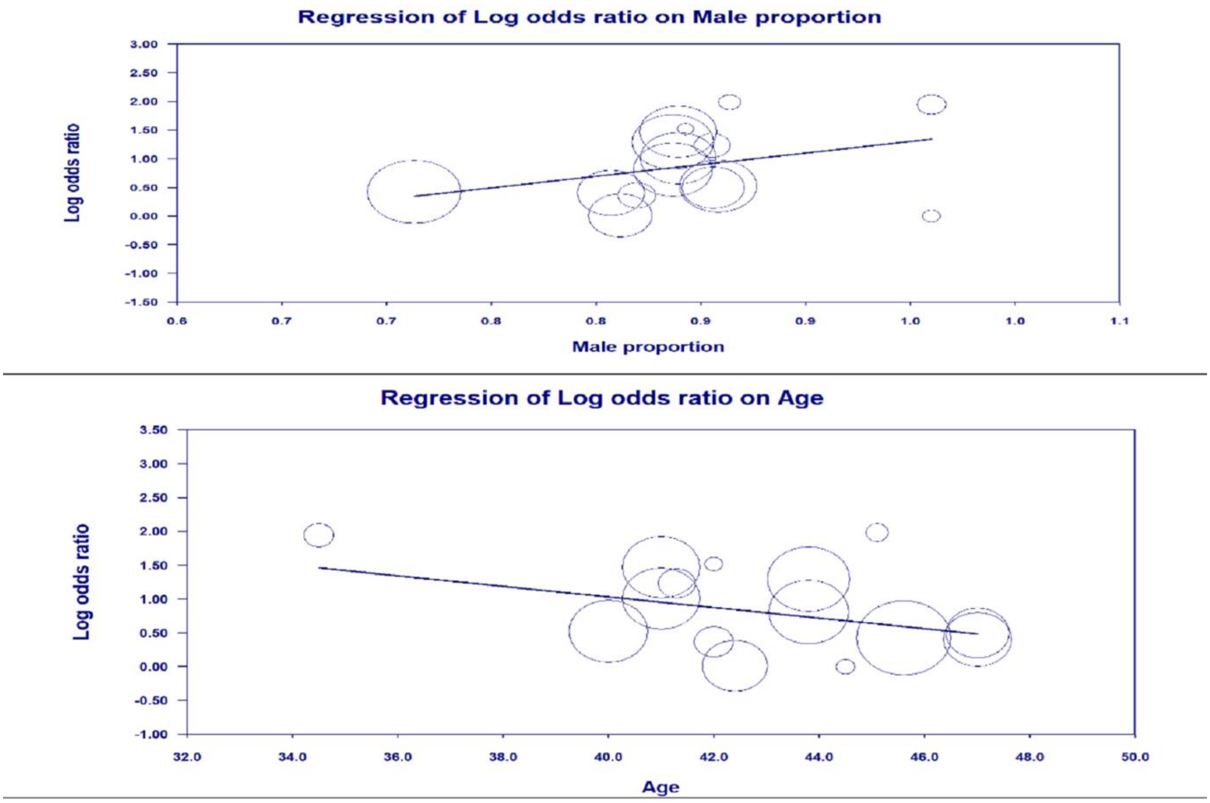

Figure S6. Meta-regression results with male proportion and age.

Table S1. The result of surface under the cumulative ranking curve (SUCRA) for frequency of attacks.

Treatment	Frequency of attacks	
	SUCRA	Rank
placebo	28	6
verapamil	59	2
melatonin	57	3
misoprostol	52	4
candesartan cilexetil	84	1
frovatriptan	18	7
cimetidine	51	5

Table S2. The result of surface under the cumulative ranking curve (SUCRA) for pain-free.

Treatment	Pain free	
	SUCRA	Rank
placebo	7	11
cortivazol	33	8
zolmitriptan 5mg	26	9
zolmitriptan 10mg	61	5
sumatriptan 6mg	70	3
lithium carbonate	19	10
betamethasone	99	1
octreotide	43	7
sumatriptan spray	52	6
capsaicin	68	4
warfarin	73	2

Table S3. The result of surface under the cumulative ranking curve (SUCRA) for duration of attacks

Treatment	Duration of attacks	
	SUCRA	Rank
placebo	56	3
valproate	9	4
misoprostol	77	1
candesartan cilexetil	58	2

Table S4. The result of surface under the cumulative ranking curve (SUCRA) for number of patients using rescue agents

Treatment	Rescue agents	
	SUCRA	Rank
placebo	4	7
zolmitriptan 5mg	32	6
zolmitriptan 10mg	41	4
sumatriptan 6mg	74	3
sumatriptan 12 mg	83	1
valproate	82	2
sumatriptan spray	35	5

Table S5. The result of surface under the cumulative ranking curve (SUCRA) for adverse events

Treatment	Adverse events	
	SUCRA	Rank
placebo	77	1
candesartan cilexetil	62	7
cortivazol	40	12
galcanezumab	63	6
zolmitriptan 5mg	51	10
zolmitriptan 10mg	38	13
sumatriptan 6mg	64	5
sumatriptan 12mg	50	11
verapamil	19	16
melatonin	76	2
valproate	58	9
lithium carbonate	27	14
misoprostol	65	4
prednisone	72	3
betamethasone	1	17
sumatriptan 100 mg	58	8
civamide	26	15
